# MSCA-Mamba: A Compact Multi-Scale Mamba Network for Multi-Label ECG Classification

**DOI:** 10.3390/diagnostics16193135

**Published:** 2026-09-26

**Authors:** Dilber Cetintas

**Affiliations:** Department of Computer Engineering, Malatya Turgut Ozal University, 44210 Malatya, Türkiye; dilber.cetintas@ozal.edu.tr

**Keywords:** electrocardiogram (ECG), multi-label classification, Mamba, Squeeze-and-Excitation (SE), parameter-efficient deep learning

## Abstract

**Objective:** Multi-label electrocardiogram (ECG) classification requires modeling morphological patterns across different temporal scales and their sequential relationships while maintaining limited model complexity. This study proposes Multi-Scale Channel-Attentive Mamba (MSCA-Mamba), a compact architecture integrating multi-scale feature extraction, branch-wise recalibration, and state-space sequence modeling. **Methods:** MSCA-Mamba combines three parallel dilated Conv1D branches, branch-wise Squeeze-and-Excitation modules, and a unidirectional Mamba block. The model was evaluated on the PTB-XL diagnostic superclass task using the official patient-wise split and five random seeds. Component ablations, parameter-matched Conv1D, GRU, LSTM, and Transformer controls, unidirectional–bidirectional comparisons, and GPU benchmarks were performed. **Results:** MSCA-Mamba achieved a Macro ROC-AUC of 0.8998 ± 0.0022, Macro PR-AUC of 0.7651 ± 0.0036, and Macro F1-score of 0.7038 ± 0.0045 with 121,149 parameters. Multi-scale feature extraction provided the clearest benefit, particularly for macro-averaged metrics. Parameter-matched alternatives showed broadly comparable performance. Bidirectional Mamba achieved a modestly higher Macro ROC-AUC but increased the parameter count to 214,077 and inference latency. **Conclusions:** MSCA-Mamba provides a compact framework for multi-label ECG classification on PTB-XL. Its primary strength lies in compact architectural integration rather than universal superiority of individual components.

## 1. Introduction

Cardiovascular diseases (CVDs) are among the leading causes of mortality and morbidity worldwide, imposing a substantial clinical and economic burden on healthcare systems [1]. Given that a considerable proportion of cardiovascular deaths result from acute events such as myocardial infarction and stroke, the early detection of cardiac abnormalities and continuous or regular monitoring of patients have become increasingly important [2]. Accordingly, the development of reliable, accessible, and cost-effective diagnostic and monitoring technologies that can facilitate the early detection of cardiovascular diseases has emerged as an important area of research. ECG is widely used in clinical practice for the assessment of cardiac conditions. As a non-invasive technique that records the electrical activity of the heart, ECG provides valuable information for the diagnosis and monitoring of cardiovascular abnormalities [3]. Alterations in cardiac electrophysiological activity are reflected in the morphology and temporal characteristics of ECG waveforms, enabling the identification of clinically relevant patterns associated with a wide spectrum of cardiac abnormalities [4]. For instance, myocardial ischemia and infarction may produce characteristic alterations in the ST segment, T wave, and QRS complex, whereas conduction disturbances and ventricular hypertrophy can also induce distinct changes in ventricular depolarization and repolarization patterns [5,6]. Importantly, ECG abnormalities are not necessarily mutually exclusive; different diagnostic findings may coexist within the same recording, reflecting the complex and heterogeneous nature of cardiac electrical activity. Consequently, automated ECG analysis requires models capable of representing heterogeneous morphological patterns while supporting multi-label diagnostic classification.

Recent systematic reviews have further highlighted the considerable potential of artificial intelligence (AI)-based ECG analysis for the diagnosis and prognosis of cardiovascular diseases [7]. Nevertheless, achieving high classification performance alone is not sufficient for the practical application of AI-based ECG classification models. For these algorithms to operate effectively under real-world conditions, additional factors such as computational complexity, memory requirements and inference latency must also be considered.

ECG classification presents a representation challenge because diagnostically relevant waveform patterns occur over different temporal scales. Conventional CNNs are effective at extracting local morphological features, but a single receptive field may not adequately represent waveform structures with different temporal extents. Multi-scale convolutional processing provides a natural mechanism for addressing this issue by extracting complementary morphological representations across different receptive fields. Beyond local morphology, sequential modeling can further be used to process relationships among the resulting temporal representations. Recurrent architectures and Transformer-based self-attention provide established solutions for sequence modeling, although their computational characteristics and parameter requirements differ substantially. State-space models such as Mamba provide an alternative formulation with linear scaling in sequence length, motivating their investigation as a compact sequential modeling mechanism following multi-scale ECG feature extraction.

Although multi-scale convolution, channel recalibration, and modern sequence-modeling mechanisms have individually been explored in ECG analysis, their integration within a compact morphology-to-sequence architecture raises an important question: can morphological information across multiple temporal scales be extracted, recalibrated, and sequentially modeled while maintaining a limited parameter budget? To investigate this question, this study proposes MSCA-Mamba, which combines parallel dilated convolutional branches, branch-wise Squeeze-and-Excitation (SE) recalibration, and unidirectional Mamba-based sequence modeling for multi-label ECG classification. Rather than assuming that each component independently improves predictive performance, the architecture is evaluated through repeated-run ablations and controlled comparisons with alternative sequence-modeling mechanisms. The main contributions of this study can be summarized as follows:

- A compact MSCA-Mamba architecture is proposed for multi-label ECG classification, integrating multi-scale dilated convolution, branch-wise SE feature recalibration, and unidirectional Mamba-based sequence modeling within a unified framework.
- A multi-scale convolutional front-end with dilation rates of 1, 2, and 4 is designed to capture ECG morphological patterns across different temporal receptive fields, with branch-wise SE recalibration applied before feature fusion.
- The architecture is systematically evaluated across five independent runs using component ablations, single- versus multi-scale and unidirectional versus bidirectional comparisons, parameter-matched sequence-modeling controls, and computational analysis.

## 2. Literature Review

The automated analysis of ECG signals has undergone a substantial methodological evolution, progressing from conventional machine learning approaches based on expert-engineered features to end-to-end deep learning architectures capable of learning hierarchical representations directly from raw data [8]. While earlier approaches were largely characterized by handcrafted features that required domain expertise and considerable effort for feature design, recent studies have increasingly focused on integrating such domain-specific knowledge with the representation-learning capabilities of modern deep learning methods [9]. Current research trends in this field can broadly be categorized into three major directions: architectural diversity, data integration strategies, and learning paradigms.

From an architectural perspective, CNNs have long been regarded as a standard approach because of their effectiveness in capturing local morphological patterns in ECG signals [10]. However, their limited ability to model long-range temporal dependencies has motivated increasing interest in self-attention mechanisms and Transformer-based architectures [11]. In this context, hybrid architectures that employ CNNs to extract local waveform characteristics while using Transformers to model global rhythmic context have gained considerable attention [12]. For example, some approaches combine multi-scale convolutional blocks with coordinate attention to capture subtle variations across temporal sequences, whereas others integrate DenseNet-based encoders with GRU- or LSTM-based decoders for automated ECG report generation [13]. In parallel, the growing demand for computational efficiency has encouraged the development of lightweight CNN architectures, such as MiniResNet, as well as optimization strategies such as integer quantization, particularly for deployment on resource-constrained edge devices [10].

With respect to data characteristics and representation, comparisons between raw time-series signals and feature-based approaches have received considerable attention in the literature [14]. Expert-engineered features have been reported to exhibit greater robustness to aggregate effects than representations learned directly from time-series data [14]. In this context, studies that augment raw ECG signals with entropy-based features or time–frequency representations, such as the discrete wavelet transform (DWT), have demonstrated improved robustness to noise [15,16]. Beyond approaches relying exclusively on ECG signals, multimodal frameworks incorporating patient demographics or clinical tabular data have been explored to improve the contextual accuracy of arrhythmia classification models [17,18]. Similarly, studies combining ECG signals with image-based information and integrating CNN and LSTM architectures through attention-based fusion mechanisms represent another important direction within this multimodal research trend [19].

The evolution of learning strategies has also been driven by the need to address the limited availability of labeled ECG data. In recent years, self-supervised learning and masked modeling techniques have gained increasing attention [20]. In particular, masked Transformer-based approaches, such as MTECG, adapt concepts from image-based autoencoding frameworks to time-series data, enabling effective representation learning from large-scale datasets [20]. Contrastive learning has also emerged as a label-efficient strategy, with applications ranging from preprocessing tasks such as noise detection to the alignment of ECG representations with clinical information [21]. In addition, weakly supervised learning techniques have been proposed to mitigate domain shifts across different datasets [22].

Despite these advances, a major limitation of existing approaches is the limited availability of the large-scale ECG datasets typically required to fully exploit Transformer-based models, together with the quadratic computational complexity inherent in self-attention mechanisms [23]. These limitations pose substantial efficiency challenges, particularly when processing long ECG sequences and deploying models on resource-constrained clinical devices [24]. Consequently, recent research has increasingly explored parameter-efficient sequence-modeling architectures, including State Space Models (SSMs) and Mamba. Through its selective state-space formulation, Mamba provides linear scaling with sequence length, offering an alternative to the quadratic scaling of conventional self-attention [25]. Mamba-based architectures have consequently been investigated in ECG analysis for classification, representation learning, and data-generation tasks [26]. These developments motivate further investigation of state-space models for ECG sequence modeling, particularly under constrained parameter budgets. Collectively, these developments highlight the potential of a new generation of lightweight yet representationally powerful models that alleviate the computational burden associated with Transformers, thereby motivating further investigation of computationally compact architectures for ECG analysis [27].

Taken together, the existing literature indicates that multi-scale convolutional feature extraction, channel attention mechanisms, and Mamba-based state-space modeling have been employed either independently or in various combinations for ECG classification. However, while a substantial proportion of existing Mamba-based ECG approaches primarily focus on improving classification performance, parameter-efficient architectures that process morphological information across multiple temporal scales through scale-specific feature recalibration and subsequently integrate these representations using a compact unidirectional Mamba block remain relatively underexplored. In particular, systematically addressing the trade-off between model size and classification performance, rather than focusing solely on performance improvements, remains an important research direction. This study addresses this gap by integrating multi-scale dilated convolutions, branch-wise SE feature recalibration, and unidirectional Mamba-based sequential modeling into a unified architecture specifically designed to operate under a limited parameter budget.

## 3. Materials and Methods

### 3.1. PTB-XL Dataset

The performance of the proposed model was evaluated using the PTB-XL dataset, which is widely used as a benchmark in ECG classification studies [28,29,30]. PTB-XL is a large-scale, publicly available dataset comprising 21,837 12-lead ECG recordings collected from 18,885 individual patients. Each recording has a duration of 10 s and was clinically annotated and labeled by up to two cardiologists. The class-wise distribution of the dataset is presented in Figure 1.

The dataset was divided into training, validation, and test sets according to the official patient-wise splitting strategy recommended by the developers of the PTB-XL dataset. Specifically, folds 1–8 were used for training, fold 9 for validation, and fold 10 as the independent test set. This patient-wise splitting strategy prevents recordings from the same patient from appearing in different data subsets, thereby minimizing the risk of data leakage between the training and evaluation stages. The classification task in this study is multi-label; therefore, a single ECG recording can be associated with more than one diagnostic superclass (NORM, MI, STTC, CD, and HYP). These classes are not mutually exclusive, and each class is predicted independently. Accordingly, the class counts reported in Table 1 represent label-wise counts rather than mutually exclusive groups.

### 3.2. Overall Architecture

The proposed architecture follows a morphology-to-sequence design in which scale-specific ECG representations are first extracted and independently recalibrated, subsequently fused, and then processed by a compact selective state-space model. As illustrated in Figure 2, the overall architecture comprises multi-scale convolutional feature extraction, branch-wise SE feature recalibration, feature fusion, Mamba-based sequential modeling, global pooling, and MLP-based classification. In this study, 12-lead ECG recordings from the PTB-XL dataset sampled at 100 Hz were used, with each recording having a duration of 10 s. Prior to being fed into the model, each lead was normalized using z-score standardization based on the mean and standard deviation calculated within the corresponding recording. No additional filtering or signal segmentation was applied, and the normalized ECG recordings were directly provided as input to the proposed model.

### 3.3. Multi-Scale Feature Extraction

The first component of the proposed architecture is the Multi-Scale Feature Extraction module. ECG signals contain morphological components that occur over different temporal durations, making it difficult to capture all relevant patterns using a single receptive field. For example, the QRS complex is characterized by short-duration, relatively high-frequency information, whereas the ST segment and T wave exhibit lower-frequency variations that extend over longer temporal intervals. Therefore, in the proposed model, the input signal is processed through parallel convolutional branches with different receptive fields to obtain a multi-scale representation.

Rather than employing different kernel sizes, a fixed kernel size (k = 7) is maintained across all branches, while only the dilation rate is varied. This design provides each branch with a comparable local filtering capacity while expanding its effective receptive field through dilation alone, thereby enabling multi-scale feature extraction without an unnecessary increase in the number of parameters. This operation is implemented within the MultiScaleChannelAttention block, where the input signal is passed through three parallel Conv1D branches. Although all branches use the same kernel size (k = 7), they employ different dilation rates, defined as d ∈ {1, 2, 4}. This configuration progressively enlarges the effective receptive field, allowing local and intermediate-scale morphological patterns to be represented simultaneously.

At a sampling frequency of 100 Hz, the branches with k = 7 and dilation rates of d = 1, d = 2, and d = 4 correspond to effective receptive fields of approximately 70 ms, 130 ms, and 250 ms, respectively. Consequently, the multi-scale front-end can capture short-duration morphological structures, such as the QRS complex, while also representing ST–T variations that require a broader temporal context. The resulting multi-scale temporal representations are subsequently processed by the Mamba block for sequential modeling. Each convolutional branch produces 32 feature maps, which are then combined along the channel dimension to form a shared feature space. This design preserves complementary morphological information extracted across different temporal scales within a unified representation. Before feature fusion, the output of each scale is independently processed by a branch-wise SE recalibration module to adaptively reweight the latent feature channels.

### 3.4. Branch-Wise SE Feature Recalibration

Although the multi-scale convolutional branches generate feature representations at different temporal scales, the latent feature channels within each branch may contribute differently to the subsequent classification process. To adaptively recalibrate these representations, an SE module is applied independently to the output of each convolutional branch. Rather than operating directly on the original ECG leads, the SE module reweights the latent feature channels generated by the convolutional filters. This branch-wise recalibration allows the contribution of individual feature channels to be adjusted within each temporal scale before feature fusion and subsequent sequential modeling.

### 3.5. Feature Fusion

Branches with smaller dilation rates are more sensitive to local morphological patterns within narrow temporal contexts, whereas branches with larger dilation rates capture broader, yet still intermediate-scale, temporal contexts. Consequently, the outputs of these branches provide complementary information, and relying on any single branch may limit the overall representational capacity of the model. To preserve the information extracted at different temporal scales, the proposed architecture combines the branch-wise features through concatenation along the channel dimension, rather than element-wise addition. This strategy retains the scale-specific information generated by each branch independently within a unified feature space, allowing subsequent layers to jointly exploit the complementary representations learned across multiple temporal scales.

F_fusion_ = *Concat* (*F*_1_,*F*_2_,*F*_3_) (1)

Here, Fi denotes the feature maps generated by the individual convolutional branches. Following feature fusion, the resulting joint representation is directly passed to the sequential modeling stage. However, because the Mamba block expects the temporal dimension to occupy the second axis of the input tensor, the feature representation produced by the Conv1D layers must first be rearranged. Specifically, the Conv1D output with dimensions [B,96,T] is transposed to [B,T,96], where B denotes the batch size and T represents the temporal dimension. More generally, the convolutional feature maps in the [B,C,T] format are transformed into the [B,T,C] representation required by the Mamba block. This rearrangement preserves the extracted feature information while ensuring compatibility with the subsequent sequential modeling operation.

### 3.6. Mamba Block

Mamba is a computationally efficient deep learning architecture based on the State Space Model (SSM) framework, designed to model temporal dependencies in long sequences with linear computational complexity [31]. In the proposed architecture, Mamba is used to process the temporal representations generated by the multi-scale convolutional front-end. While the convolutional branches extract morphological features across different receptive fields, the Mamba block provides a state-space-based mechanism for modeling sequential relationships among the resulting temporal representations.

Accordingly, the Mamba block is positioned after the multi-scale feature extraction, branch-wise SE recalibration, and feature-fusion stages in the proposed architecture. The fused multi-scale feature representations are passed to the Mamba block for sequential modeling along the temporal dimension. In this design, convolution-based morphological feature extraction and state-space-based sequential modeling are organized as successive and complementary stages, allowing the model to integrate multi-scale morphological representations with their temporal relationships.

Before being passed to the Mamba block, the input features are processed using Layer Normalization. Since the multi-scale convolutional branches extract features from different temporal receptive fields, the activation distributions of the resulting channels may vary after feature fusion. Layer Normalization normalizes the feature distribution at each time step, enabling representations derived from different temporal scales to be presented to the Mamba block in a more balanced manner. This operation also limits variations in activation magnitude, thereby contributing to a more stable optimization process. In particular, applying normalization before sequential modeling allows the Mamba block to process the input features within a more consistent representation space. The normalized features are subsequently passed to a unidirectional Mamba layer. Based on the Selective State Space framework, the Mamba module updates the state information at each time step while maintaining information flow throughout the sequence with linear computational complexity. The sequence of operations within the Mamba block is defined by the following equations:

x_2_ = LN(x_1_) (2)

H = Mamba(x_2_) (3)

H = Dropout(H)(4)

Y = x_1_ + H (5)

O = LN_final_(Y) (6)

Here, LN(⋅) denotes Layer Normalization, H represents the intermediate representation generated by the Mamba layer, and Y denotes the output of the residual Mamba block, whereas O denotes the representation obtained after the separate final Layer Normalization. The Dropout operation applied after the Mamba layer contributes to reducing overfitting by randomly deactivating a subset of activations during training. The residual addition completes the unidirectional Mamba block. No second Layer Normalization is applied within the block. After the complete Mamba stack, a separate final Layer Normalization is applied before the subsequent global pooling stage, as expressed in Equation (6). The main hyperparameters of the Mamba layer were set to dstate = 16, dconv = 4, and expand = 2. While Transformer-based self-attention mechanisms exhibit quadratic computational complexity with respect to sequence length, Mamba provides a state-space formulation with linear scaling in sequence length. Accordingly, Mamba was investigated in the proposed architecture as a compact sequential modeling mechanism for processing the temporal representations generated by the multi-scale convolutional front-end.

### 3.7. Global Pooling and Classification

The output of the Mamba block is passed through parallel Global Average Pooling (GAP) and Global Max Pooling (GMP) operations to transform the temporal feature sequence into a fixed-dimensional representation. GAP and GMP are independently applied to the same feature sequence. While GAP summarizes activations that are consistently distributed across the temporal dimension, GMP preserves the strongest discriminative responses occurring at any point in the sequence. It should be noted that, although the Mamba block operates unidirectionally, the current architecture is not designed for causal or streaming inference. Since the GAP and GMP operations aggregate information over the complete temporal sequence, the final classification requires the entire 10 s ECG recording to be available. Therefore, in the present architecture, the practical benefit of unidirectional Mamba lies primarily in reduced computational complexity relative to the bidirectional configuration, rather than in enabling online processing.

These complementary representations are subsequently concatenated along the feature dimension. As a result, the two 96-dimensional vectors are combined to form a final 192-dimensional feature representation. The resulting 192-dimensional feature vector is then passed to a Multi-Layer Perceptron (MLP)-based classifier to generate the final class predictions. Since the proposed architecture is designed for multi-label classification, no activation function is applied directly at the output layer. Instead, the output logits are optimized using BCEWithLogitsLoss during training, which internally incorporates the sigmoid transformation and thereby improves numerical stability. During inference, the logits are converted into class probabilities using the sigmoid function, and the final class labels are determined according to predefined decision thresholds. To ensure that threshold selection remains independent of the test set, class-specific decision thresholds are determined exclusively using the validation set. For each diagnostic class, a threshold search is performed over the range of 0.05–0.95 with increments of 0.01, and the threshold that maximizes the validation F1-score is selected. The resulting class-specific thresholds are subsequently applied to the independent test set without any further optimization. Threshold-independent evaluation metrics, including ROC-AUC and PR-AUC, are calculated directly from the predicted class probabilities.

## 4. Experimental Results

All retained model configurations were evaluated across five independent training runs using the random seeds 42, 123, 456, 789, and 2025. Because the same seed set was used across model configurations, architecture-level comparisons were additionally performed using seed-wise paired differences. For each comparison, the mean paired difference and the corresponding two-sided 95% t confidence interval across the five paired runs were calculated. Given the limited number of runs (n = 5), these confidence intervals were interpreted as descriptive estimates of training variability rather than definitive hypothesis tests. All experiments followed the patient-wise data-splitting protocol recommended for the PTB-XL dataset, and the proposed model and all retained ablation variants were trained and evaluated under identical experimental conditions. The models were trained for a maximum of 30 epochs using the AdamW optimizer with an initial learning rate of 1 × 10^−4^, a weight decay of 1 × 10^−4^, and a batch size of 16. Unweighted BCEWithLogitsLoss was used for multi-label classification; no class weighting or resampling strategy was applied during training. Early stopping was based on the validation Macro ROC-AUC, with a patience of 7 epochs and a minimum improvement threshold (min_delta) of 1 × 10^−4^. The model checkpoint achieving the highest validation Macro ROC-AUC was retained for final evaluation. For each independent run, class-specific decision thresholds were re-selected exclusively on the validation set according to the procedure described in Section 3.7 and were then fixed for test-set evaluation. Results across the five runs are reported as mean ± sample standard deviation.

In this section, the performance of the proposed MSCA-Mamba model on the independent test set is analyzed in detail. The model was evaluated using ROC-AUC, PR-AUC, F1-score, precision, recall, and subset accuracy. ROC-AUC quantifies the model’s ability to discriminate between classes, whereas PR-AUC characterizes performance in terms of the precision–recall relationship. Precision measures the proportion of positive predictions that are correct, recall reflects the ability of the model to identify positive instances, and the F1-score provides a harmonic balance between precision and recall. In the context of multi-label classification, subset accuracy represents the proportion of samples for which the complete set of predicted labels exactly matches the corresponding ground-truth label set. In addition to these quantitative metrics, ROC curves, precision–recall curves, and class-wise confusion matrices were analyzed to provide a more detailed assessment of the model’s discriminative performance across individual diagnostic classes.

Table 2 summarizes the test-set performance of the proposed MSCA-Mamba across five independent training runs using random seeds 42, 123, 456, 789, and 2025. Results are reported as the mean ± sample standard deviation across the five runs. MSCA-Mamba achieved a mean Macro ROC-AUC of 0.8998 ± 0.0022 and a mean Micro ROC-AUC of 0.9119 ± 0.0052. The corresponding Macro PR-AUC was 0.7651 ± 0.0036, while the Macro and Micro F1-scores were 0.7038 ± 0.0045 and 0.7474 ± 0.0074, respectively. The model further achieved a Weighted F1-score of 0.7494 ± 0.0039 and a subset accuracy of 0.5691 ± 0.0143.

Figure 3 illustrates the training and validation trajectories for a representative training run of MSCA-Mamba. The validation Macro ROC-AUC increased rapidly during the early epochs and subsequently stabilized near 0.90, while the Macro F1-score followed a similar pattern and stabilized near 0.70. These curves are presented as a descriptive illustration of the optimization behavior of a single run.

To place the performance of MSCA-Mamba in the context of previous ECG classification studies, Table 3 summarizes selected literature-reported methods evaluated on the PTB-XL diagnostic classification task. Classification performance and the number of trainable parameters are reported where available.

In Table 3, MSCA-Mamba achieved a mean Macro ROC-AUC of 0.8998 ± 0.0022 across five runs, while some previously published methods reported higher values. However, direct comparison remains limited by differences in preprocessing, evaluation protocols, and metric definitions across studies. Therefore, Table 3 is presented to provide literature context.

### 4.1. Class-Wise Performance Analysis

When the model performance is evaluated on a class-wise basis, noticeable variations can be observed across different cardiac diagnostic categories. Figure 4 presents the ROC-AUC, PR-AUC, and F1-score achieved by the proposed MSCA-Mamba model for each diagnostic superclass, providing a clear visual comparison of class-wise performance.

In the seed-42 run, ROC-AUC values were 0.9394, 0.9142, 0.9305, 0.9144, and 0.8115 for NORM, MI, STTC, CD, and HYP, respectively. NORM also showed the highest F1-score (0.8538), whereas HYP showed the lowest PR-AUC (0.4662) and F1-score (0.4682). These results illustrate that classification performance varied across the diagnostic superclasses, with HYP being comparatively more difficult to identify in this representative run. To further examine these class-wise performance differences, Figure 5 presents the corresponding confusion matrices, providing a more detailed view of the distribution of correct and incorrect predictions for each diagnostic superclass.

Figure 5 presents the class-wise confusion matrices for the representative seed-42 run. NORM showed the highest recall (0.9335), followed by MI (0.7855) and STTC (0.7774). CD exhibited lower recall (0.6714), mainly due to a higher number of false-negative predictions. HYP was the most challenging class, with 129 true-positive and 133 false-negative predictions, corresponding to a recall of 0.4924.

### 4.2. Ablation Study

A comprehensive ablation study was conducted to evaluate the contributions of the main architectural components of MSCA-Mamba. Using the proposed model as the reference configuration, branch-wise SE recalibration and Mamba-based sequence modeling were first evaluated through component-removal experiments. The effects of single-scale versus multi-scale feature extraction and unidirectional versus bidirectional Mamba modeling were also examined. In addition, parameter-matched Conv1D, GRU, LSTM, and Transformer models were evaluated to compare Mamba with alternative sequence-modeling approaches under similar model capacities. All retained configurations were evaluated using the same data split, training protocol, hyperparameters, and five random seeds. The corresponding component-ablation and SE-placement ablation results are presented in Table 4 and Table 5, respectively.

As shown in Table 4, removing SE recalibration or the Mamba block resulted in only small and inconsistent changes in performance. In contrast, the multi-scale configuration achieved higher Macro ROC-AUC, Macro PR-AUC, and Macro F1 than the single-scale variant, with paired 95% confidence intervals excluding zero for these metrics. However, the improvement was not observed across all metrics. The multi-scale model also required 6336 additional parameters (5.52%). Overall, these results suggest a modest benefit of multi-scale feature extraction, particularly for macro-averaged performance.

Table 5 evaluates the effect of SE placement within the proposed architecture. No single placement consistently outperformed the others across all metrics. Branch-wise SE achieved the highest Macro PR-AUC (0.7693), whereas post-fusion SE yielded the highest Macro ROC-AUC (0.9028) and post-Mamba SE achieved the highest Macro F1-score (0.7140). The branch-wise configuration was retained in MSCA-Mamba because it enables scale-specific channel recalibration before feature fusion, consistent with the intended multi-scale feature-processing design, rather than because it demonstrated universal performance superiority. Following the SE-placement analysis, we next evaluated the Mamba-based sequence-modeling component against parameter-matched alternatives. The corresponding results across five independent runs are presented in Table 6.

Under the same experimental protocol and a closely matched parameter budget, the Conv1D, GRU, and LSTM alternatives achieved higher mean Macro ROC-AUC than MSCA-Mamba, whereas the Transformer achieved a lower mean value. The paired mean differences in Macro ROC-AUC relative to MSCA-Mamba were +0.0045 for Conv1D, +0.0064 for GRU, +0.0046 for LSTM, and −0.0026 for Transformer. The corresponding 95% confidence intervals excluded zero. Building on the parameter-matched comparisons in Table 6, we further investigated whether incorporating bidirectional temporal context alters the performance and complexity of the Mamba-based architecture. Accordingly, Table 7 compares the unidirectional and bidirectional Mamba configurations across five independent runs.

The bidirectional configuration achieved a higher mean Macro ROC-AUC than the unidirectional MSCA-Mamba (0.9047 vs. 0.8998), with a paired mean difference of +0.0050 and a 95% confidence interval excluding zero. However, the bidirectional configuration increased the parameter count from 121,149 to 214,077. These results indicate a modest Macro ROC-AUC benefit from bidirectional modeling at the cost of substantially increased model complexity. To complement the performance and model-complexity analysis in Table 7, we further evaluated the computational characteristics of MSCA-Mamba and the alternative sequence-modeling configurations under a common GPU benchmarking setting. Table 8 reports their parameter count, model size, inference latency, throughput, and peak GPU memory usage under a common benchmarking setting.

### 4.3. Computational Complexity Analysis

As shown in Table 8, MSCA-Mamba provides a compact implementation with 121,149 trainable parameters and only 0.462 MB of FP32 parameter storage. On the NVIDIA L4 GPU, it achieved a mean inference latency of 2.286 ms per recording and a P95 latency of 2.356 ms. The value of this compact design is particularly evident when compared with the bidirectional variant: the unidirectional model reduces the parameter count from 214,077 to 121,149 while also reducing mean inference latency from 2.904 to 2.286 ms. At the same time, the parameter-matched Conv1D, GRU, LSTM, and Transformer alternatives achieved lower mean latency, showing that the observed compactness should not be interpreted as a Mamba-specific speed advantage. These results therefore highlight the small computational footprint of MSCA-Mamba while providing a more balanced view of its efficiency relative to alternative sequence models. Since all measurements were obtained on an NVIDIA L4 GPU, they represent relative computational characteristics under a common hardware setting rather than practical edge-device performance. To complement the quantitative results in Table 8, Figure 6 and Figure 7 visualize the inference latency and throughput of the evaluated models, respectively.

Figure 6 compares the mean inference latency of the evaluated models using the same GPU inference setting. MSCA-Mamba achieved a mean latency of 2.286 ms per recording. The parameter-matched Conv1D, GRU, LSTM, and Transformer alternatives exhibited lower mean latency, ranging from 1.843 to 2.061 ms per recording. These results show that the compact parameter count of MSCA-Mamba does not translate into the lowest inference latency among the evaluated architectures.

As shown in Figure 7, MSCA-Mamba processed 437.48 records/s. The parameter-matched Conv1D, GRU, LSTM, and Transformer alternatives achieved higher throughput, ranging from 485.09 to 542.46 records/s, with the matched Conv1D model reaching 542.46 records/s. These findings are consistent with the latency measurements.

## 5. Discussion

This study investigated whether multi-scale morphological feature extraction, branch-wise channel recalibration, and Mamba-based sequential modeling can be integrated within a compact architecture for multi-label ECG classification. Beyond evaluating the proposed MSCA-Mamba architecture itself, the experiments were designed to examine the contribution of its individual components, the effect of multi-scale feature extraction, the influence of unidirectional versus bidirectional Mamba modeling, and the behavior of Mamba relative to parameter-matched sequence-modeling alternatives. The resulting findings provide a more nuanced view of the architecture, showing that the observed performance cannot be attributed uniformly to all of its individual components.

Across five independent training runs, MSCA-Mamba achieved a mean Macro ROC-AUC of 0.8998 ± 0.0022, Micro ROC-AUC of 0.9119 ± 0.0052, Macro PR-AUC of 0.7651 ± 0.0036, and Macro F1-score of 0.7038 ± 0.0045. The relatively small standard deviation in Macro ROC-AUC indicates limited variation in ranking performance across the evaluated seeds, although greater variability was observed for some threshold-dependent metrics.

Class-wise analysis further showed that performance was not uniform across the five diagnostic superclasses. In the representative seed-42 run, NORM achieved the highest F1-score (0.8538), whereas HYP showed the lowest PR-AUC (0.4662) and F1-score (0.4682). The corresponding confusion matrices showed a recall of 0.4924 for HYP, with 129 true-positive and 133 false-negative predictions. In contrast, NORM achieved a recall of 0.9335. These findings indicate that the model had greater difficulty identifying HYP than the other diagnostic superclasses in this representative run.

Among the evaluated architectural components, multi-scale feature extraction showed the clearest positive effect on macro-averaged performance. Relative to the single-scale variant, the multi-scale MSCA-Mamba increased mean Macro ROC-AUC by 0.00336, Macro PR-AUC by 0.00719, and Macro F1 by 0.00612. The corresponding paired 95% confidence intervals excluded zero for these metrics. However, the improvement was not uniform across all evaluation measures; for example, the paired confidence intervals for Micro ROC-AUC, Micro F1, and subset accuracy included zero. Moreover, the multi-scale configuration required 6336 additional trainable parameters, corresponding to a 5.52% increase over the single-scale model. These findings therefore support a modest benefit of multi-scale feature extraction, particularly for macro-averaged performance, rather than a universal improvement across all metrics. In contrast, the standalone contribution of branch-wise SE recalibration was small and sensitive to training variability. Removing the SE modules produced only minor changes in the mean evaluation metrics, and the paired 95% confidence interval for the Macro ROC-AUC difference between MSCA-Mamba and its no-SE variant included zero. Similar uncertainty was observed for the other evaluated metrics. Thus, although branch-wise SE provides a mechanism for scale-specific channel recalibration before feature fusion, the present experiments do not establish a consistent classification-performance benefit from this component alone. Its primary role in the proposed architecture is to recalibrate the latent feature channels extracted at each temporal scale before feature fusion, rather than to serve as an independently validated source of performance improvement.

Under closely matched parameter budgets, the Conv1D, GRU, and LSTM alternatives achieved higher mean Macro ROC-AUC than MSCA-Mamba, whereas the Transformer achieved a lower mean value. Importantly, MSCA-Mamba maintained competitive classification performance while preserving a compact parameter budget of approximately 121 k parameters. These findings suggest that Mamba represents a viable state-space sequence-modeling choice for processing the temporal representations produced by the multi-scale convolutional front-end, although the present experiments do not demonstrate a universal performance advantage over conventional alternatives. Therefore, the contribution of Mamba in the proposed architecture lies in enabling compact state-space sequential modeling within the integrated MSCA framework, rather than in claiming superiority over other sequence-modeling mechanisms.

The comparison between unidirectional and bidirectional Mamba configurations provides further insight into this design choice. BiMamba achieved a higher mean Macro ROC-AUC than the unidirectional MSCA-Mamba (0.9047 ± 0.0018 versus 0.8998 ± 0.0022), with a paired mean difference of +0.0050 and a 95% confidence interval of [0.0004, 0.0096]. Moreover, bidirectional modeling increased the number of trainable parameters from 121,149 to 214,077. Thus, bidirectional processing provided a modest Macro ROC-AUC improvement under the evaluated setting, but this improvement was accompanied by a substantially larger parameter footprint. The unidirectional configuration was therefore retained as the proposed architecture to preserve the compact design objective, rather than because it demonstrated superior classification performance.

From a computational perspective, MSCA-Mamba maintained a compact model size, with 121,149 trainable parameters and approximately 0.462 MB of FP32 parameter storage. The unidirectional model also required substantially fewer parameters than its bidirectional counterpart (214,077 parameters), consistent with the compact-design objective of the proposed architecture. However, parameter count did not directly translate into lower inference latency. On the NVIDIA L4 GPU, MSCA-Mamba achieved a mean latency of 2.286 ms and a throughput of 437.48 records/s, whereas the parameter-matched Conv1D, GRU, LSTM, and Transformer alternatives exhibited lower mean latencies and higher throughput. These results indicate that the main computational characteristic of MSCA-Mamba is its small parameter footprint rather than a Mamba-specific inference-speed advantage.

Direct numerical comparison across studies is inherently limited by differences in preprocessing, label construction, training protocols, threshold selection, and evaluation settings. Instead, the present study focuses on integrating multi-scale morphological feature extraction, branch-wise channel recalibration, and state-space sequence modeling within a compact architecture. With approximately 121 k trainable parameters, MSCA-Mamba demonstrates that this combination can be implemented with a small parameter footprint while maintaining robust multi-label classification performance across repeated training runs.

Despite these findings, the use of unidirectional Mamba also represents a potential limitation for offline ECG classification, where the complete recording is available and information from both temporal directions may provide complementary context. In the present experiments, BiMamba achieved a modestly higher Macro ROC-AUC than the unidirectional configuration, but this improvement was accompanied by a substantial increase in model size and inference latency. Thus, the choice of unidirectional Mamba in MSCA-Mamba primarily reflects the compact-design objective rather than superior predictive performance. Moreover, because the global pooling stage requires the complete 10-s ECG sequence, the current architecture should not be interpreted as a causal or streaming model. A further limitation concerns the computational evaluation: all inference benchmarks were conducted on an NVIDIA L4 GPU under a common experimental setting. Therefore, the reported latency, throughput, and memory measurements characterize relative computational behavior among the evaluated models rather than practical efficiency on embedded or wearable hardware. Future work should investigate more parameter-efficient bidirectional or context-aware state-space formulations and evaluate their generalizability and computational behavior across different ECG datasets, diagnostic tasks, and resource-constrained hardware platforms.

## 6. Conclusions

This study presented MSCA-Mamba, a compact architecture for multi-label ECG classification that integrates multi-scale convolutional feature extraction, branch-wise channel recalibration, and Mamba-based state-space sequence modeling. Across five independent runs on the PTB-XL diagnostic superclass task, the proposed model achieved a Macro ROC-AUC of 0.8998 ± 0.0022, Macro PR-AUC of 0.7651 ± 0.0036, and Macro F1-score of 0.7038 ± 0.0045 with 121,149 trainable parameters.

Controlled ablation experiments showed that multi-scale feature extraction provided the clearest benefit, particularly for macro-averaged metrics, whereas the standalone contribution of SE recalibration was comparatively small and variable across seeds. Parameter-matched comparisons further demonstrated that Mamba provides a viable compact mechanism for sequential modeling within the proposed framework, while its performance remained broadly comparable to the evaluated conventional alternatives and did not indicate a universal advantage across metrics. Bidirectional Mamba yielded a modest improvement in Macro ROC-AUC but substantially increased model size and inference latency. Overall, these findings establish MSCA-Mamba as a compact and experimentally characterized framework for multi-label ECG classification, combining multi-scale morphological feature extraction with state-space sequence modeling while maintaining a small parameter footprint and stable performance across repeated runs.

## Figures and Tables

**Figure 1:**
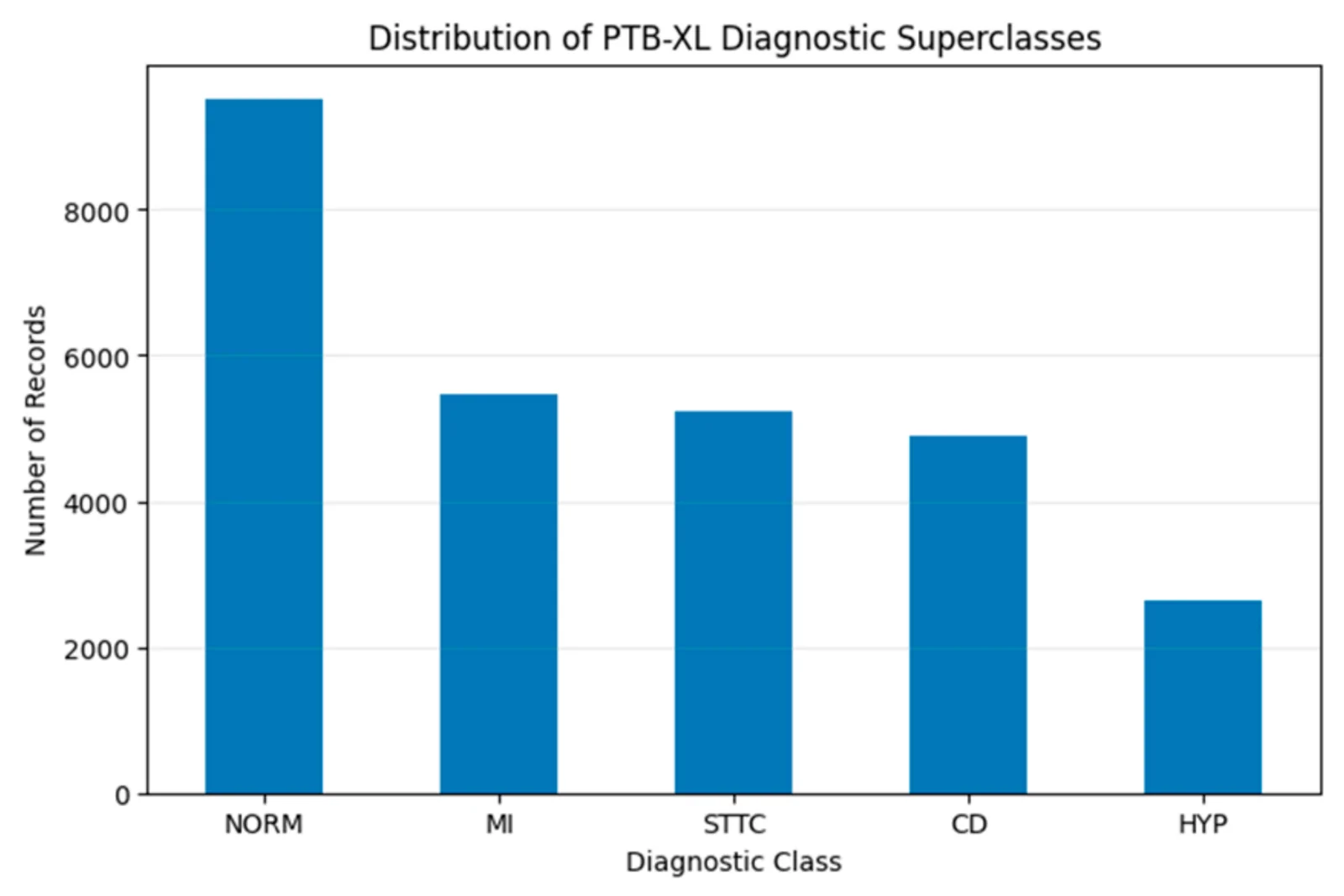
Class-wise distribution of ECG recordings in the PTB-XL dataset.

**Figure 2:**
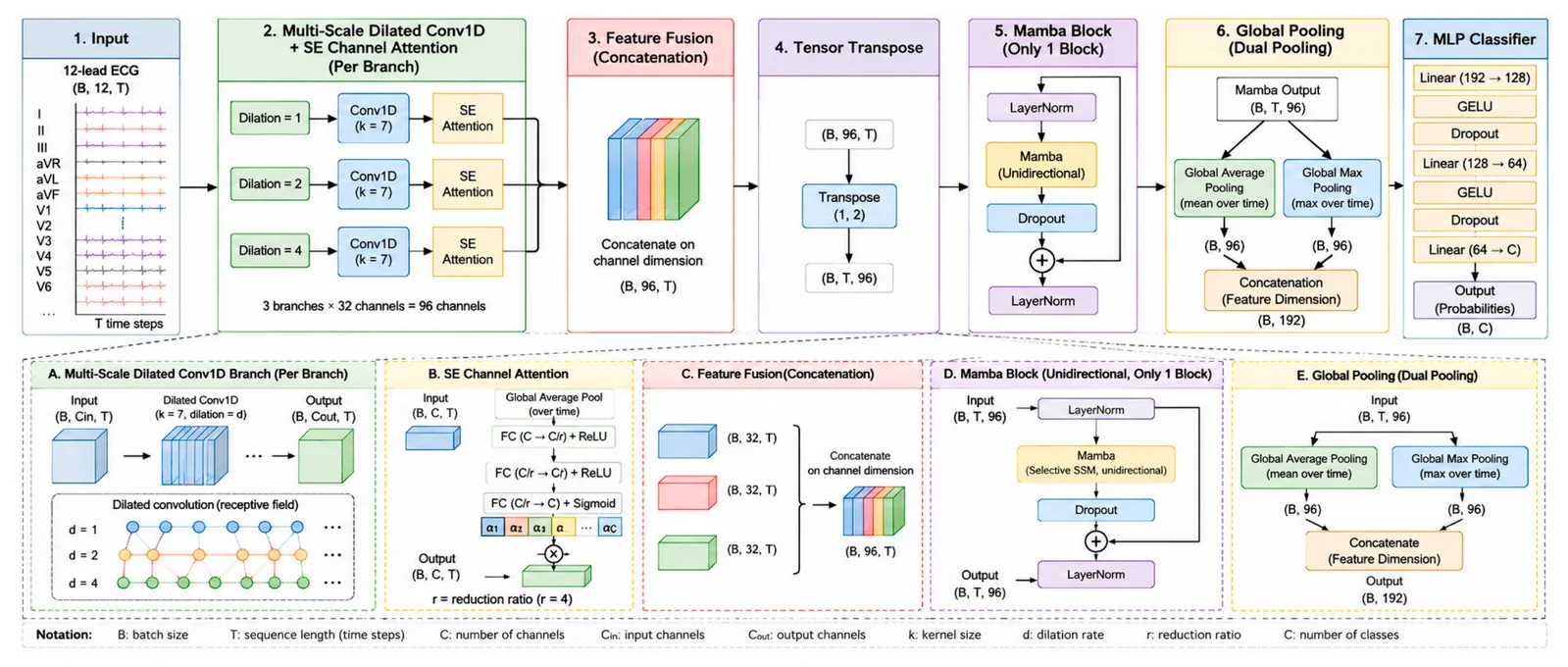
MSCA-Mamba Architecture.

**Figure 3:**
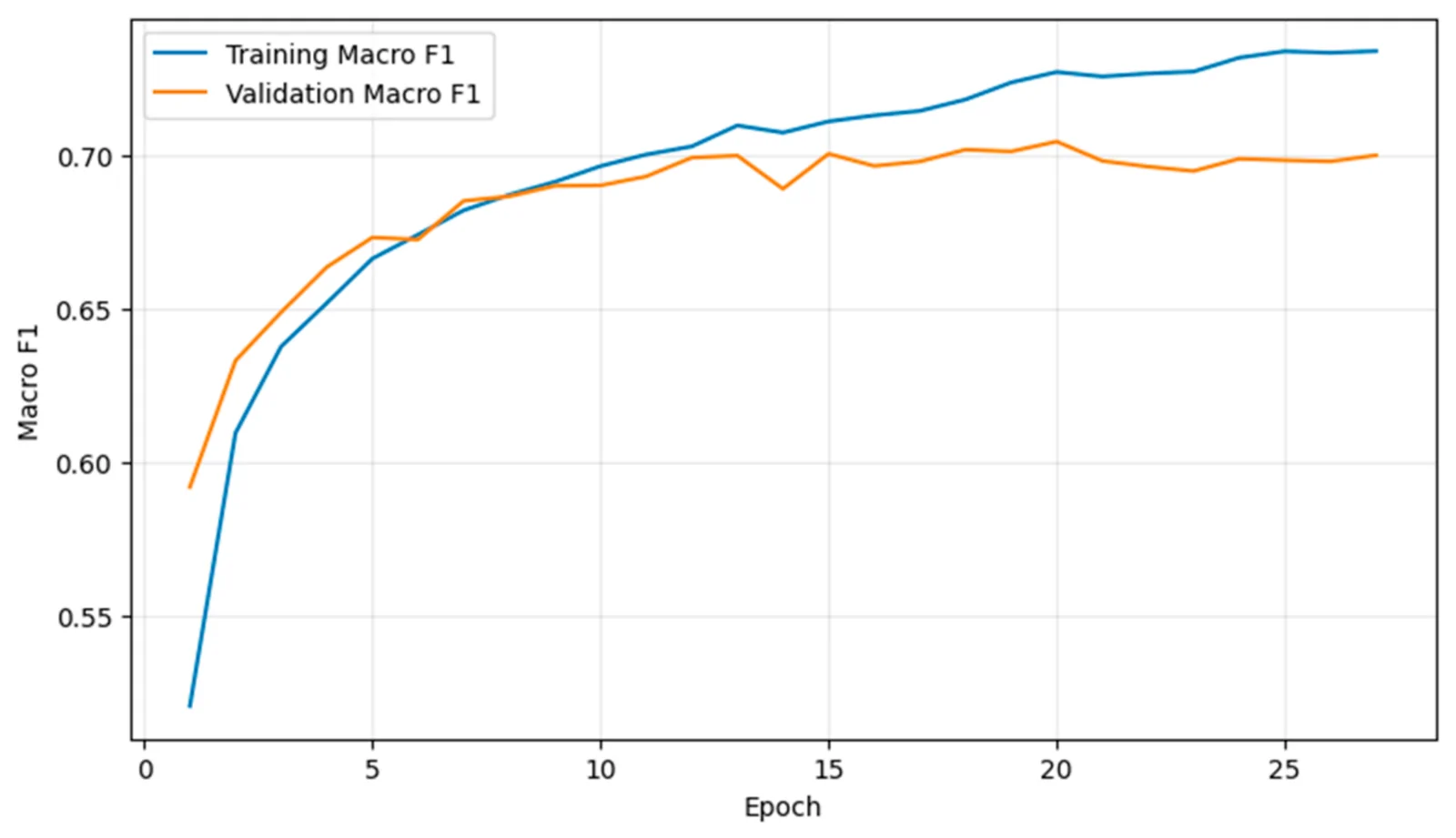
Training and validation performance curves of the proposed model in terms of Macro ROC-AUC and Macro F1-score.

**Figure 4:**
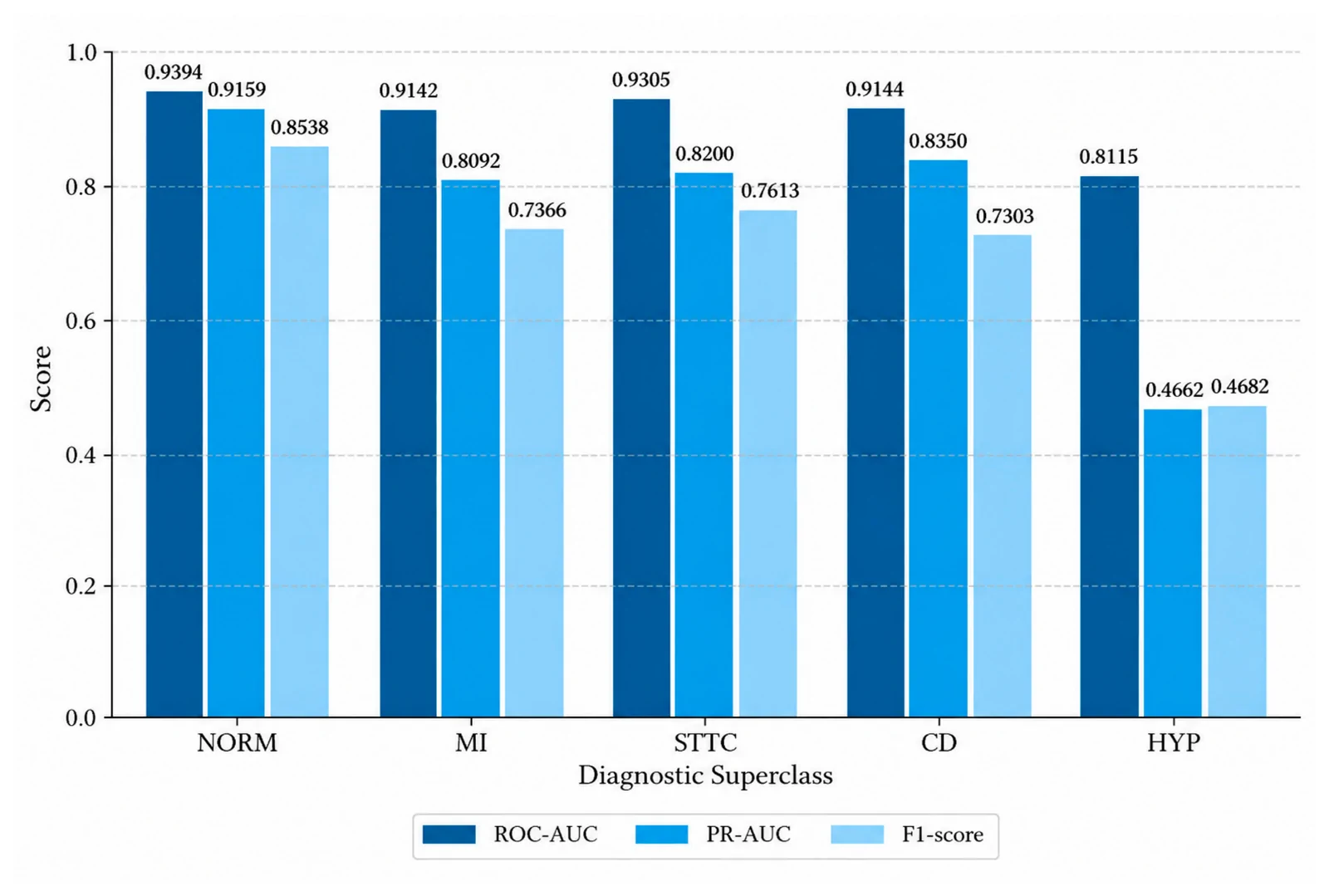
Class-wise performance of the proposed MSCA-Mamba model on the PTB-XL test set in terms of ROC-AUC, PR-AUC, and F1-score (seed = 42).

**Figure 5:**
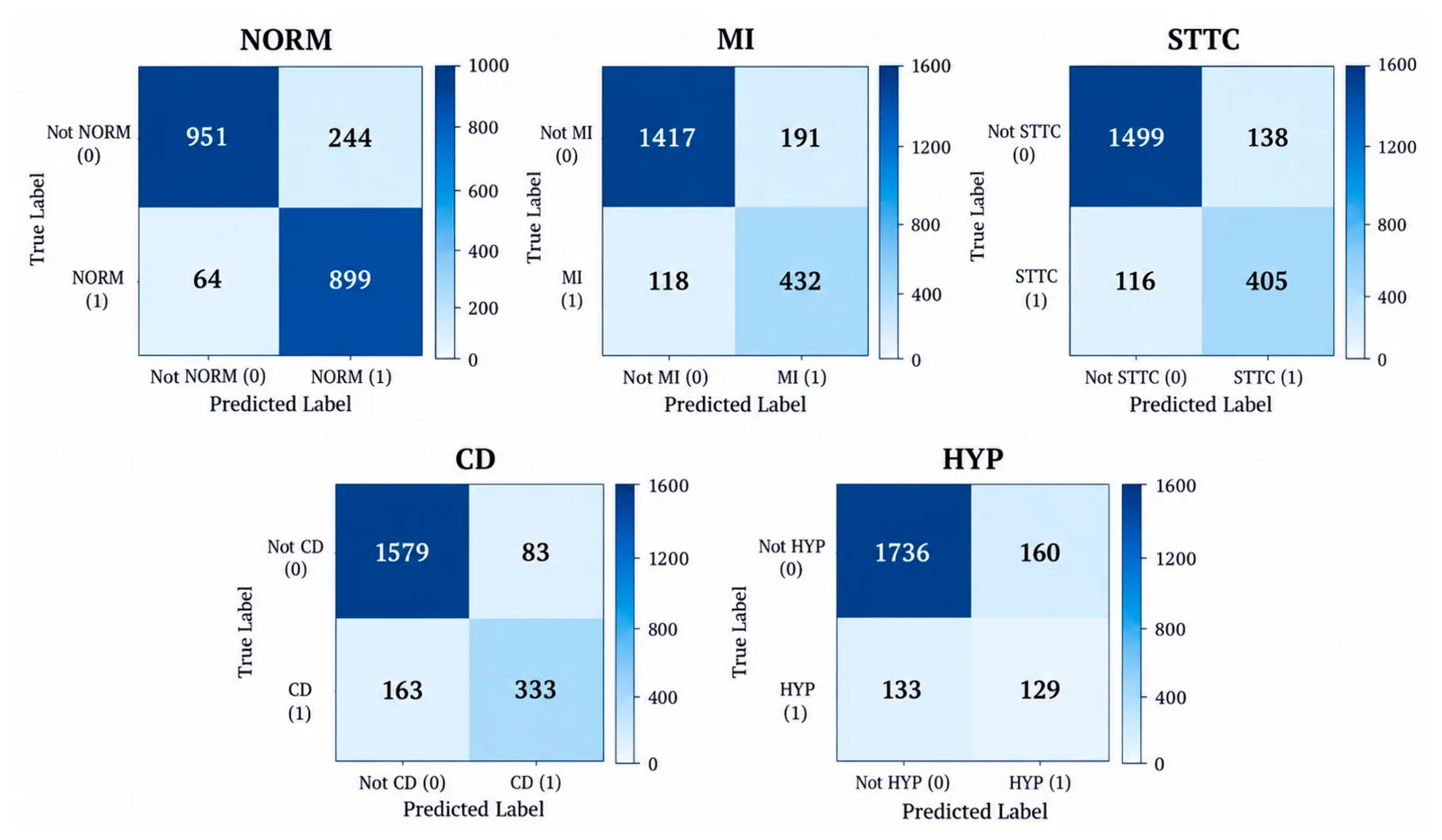
Class-wise confusion matrices of the MSCA-Mamba model on the PTB-XL test set.

**Figure 6:**
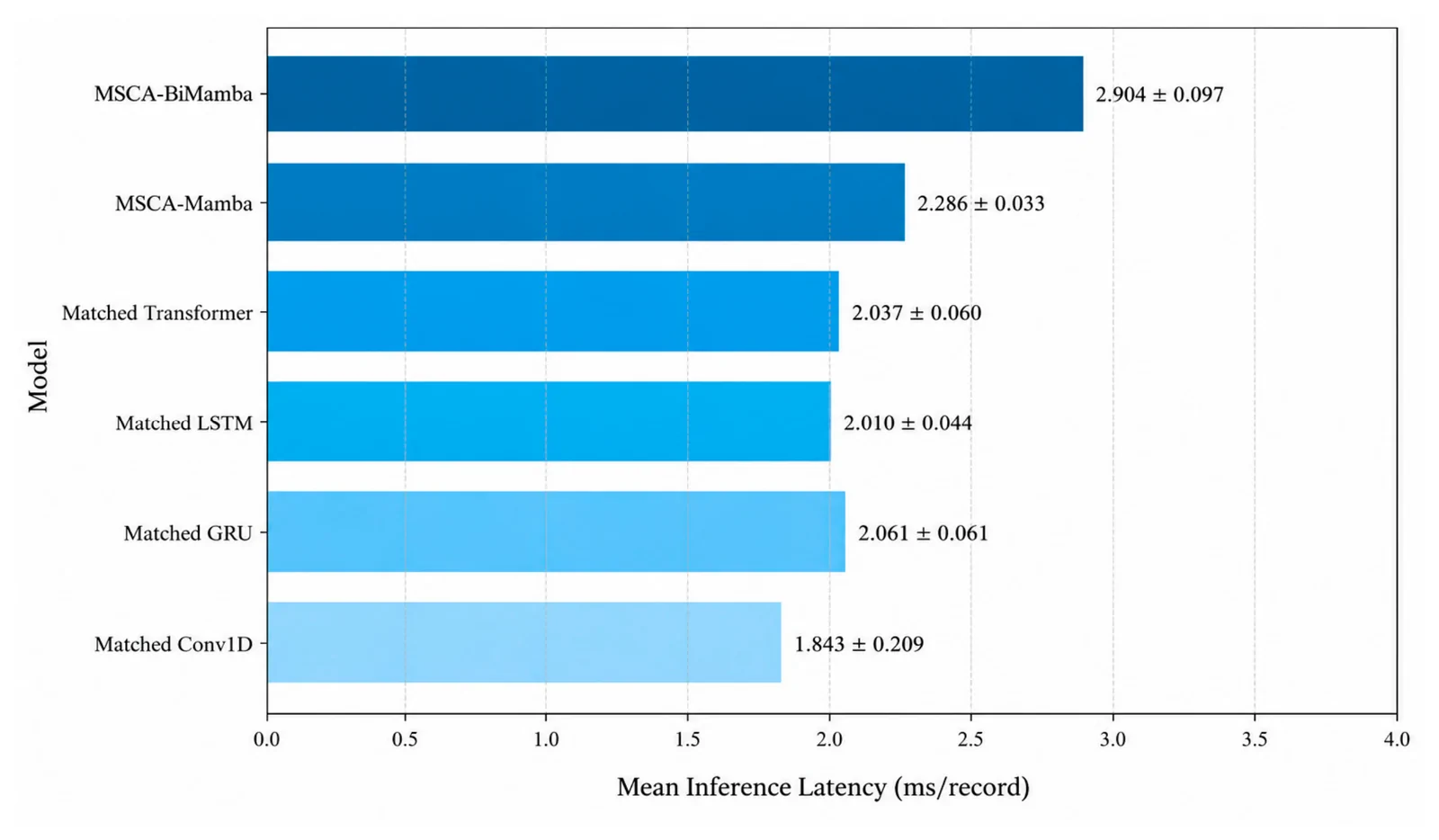
Inference latency comparison of the evaluated models.

**Figure 7:**
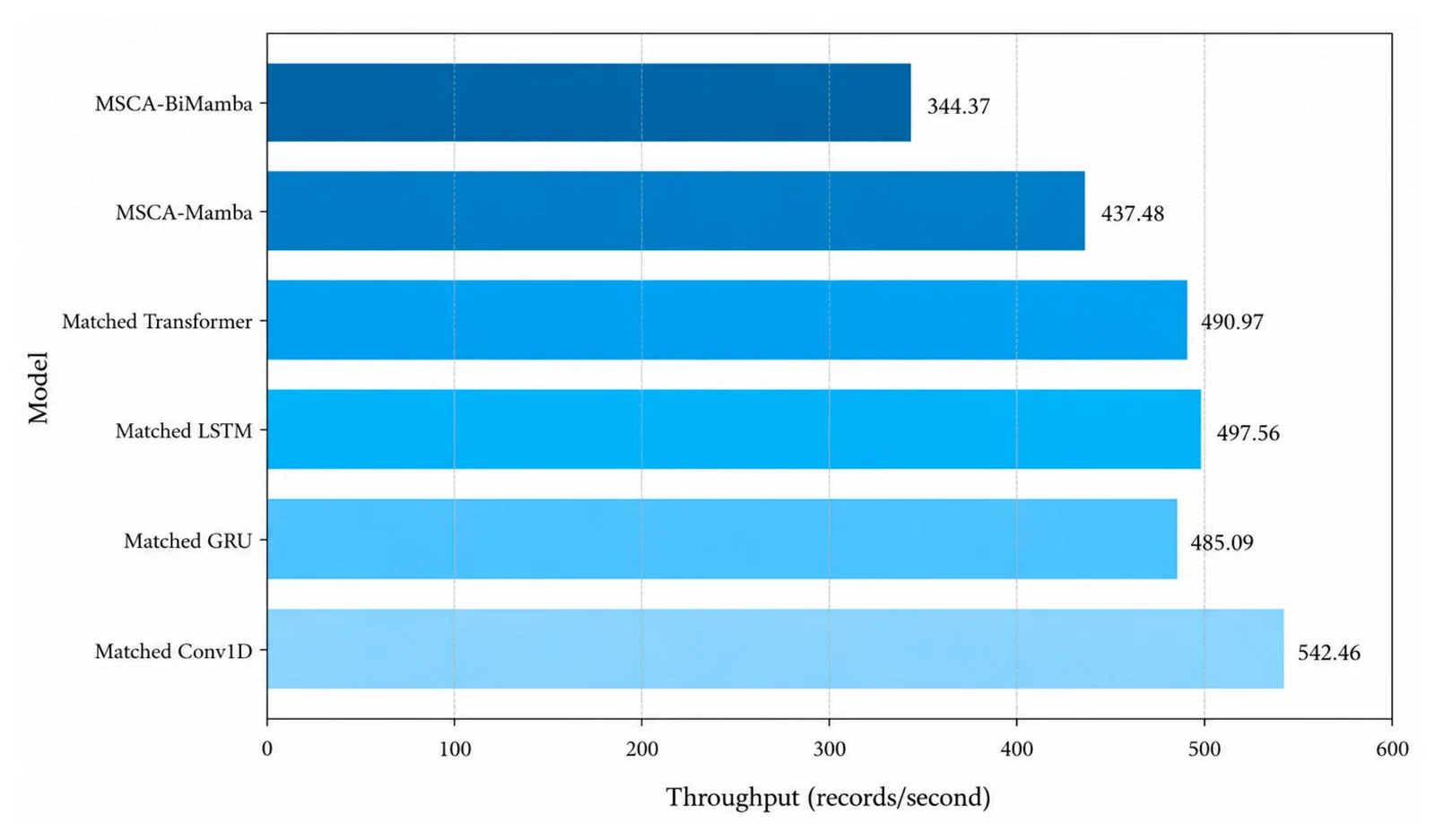
Inference throughput comparison of the evaluated models.

**Table 1:** Label-wise distribution of the five diagnostic superclasses across the training, validation, and test sets.

Class	Training Count	Training (%)	Validation Count	Validation (%)	Test Count	Test (%)
**NORM**	7596	44.46	955	44.50	963	44.62
**MI**	4379	25.63	540	25.16	550	25.49
**STTC**	4186	24.50	528	24.60	521	24.14
**CD**	3907	22.87	495	23.07	496	22.98
**HYP**	2119	12.40	268	12.49	262	12.14

**Table 2:** Overall test-set performance of the proposed MSCA-Mamba across five independent training runs.

Model	Macro ROC-AUC	Micro ROC-AUC	Macro PR-AUC	Macro F1	Micro F1	Weighted F1	Subset Accuracy
**MSCA-Mamba**	0.8998 ± 0.0022	0.9119 ± 0.0052	0.7651 ± 0.0036	0.7038 ± 0.0045	0.7474 ± 0.0074	0.7494 ± 0.0039	0.5691 ± 0.0143

**Table 3:** Overview of literature-reported results on the PTB-XL diagnostic classification task.

Reference	Method	PTB-XL Task	Results	Params
**Strodthoff et al. (2020)—ResNet-based 1D CNN** [9]	1D Residual CNN	Diagnostic superclasses	Macro ROC-AUC: 0.930	-
**Reddy et al. (2021)—** **IMLE-Net** [32]	Multi-level Multi-channel Attention Network	Diagnostic superclasses	Macro ROC-AUC: 0.9216	-
**Cheng et al. (2023)—** **MSW-Transformer** [33]	Multi-Scale Shifted-Window Transformer	Diagnostic superclasses	average macro-F1: 77.85	-
**Qiang et al. (2024)—** **ECGMamba** [34]	Bidirectional Selective State Space Model (BiSSM/Mamba)	Diagnostic superclasses	AUC: 0.9167	5.31 M
**MSCA-Mamba** **(Proposed)**	Multi-Scale Dilated Conv1D + Branch-Wise SE Recalibration + Unidirectional Mamba	Diagnostic superclasses	Macro ROC-AUC: 0.8998 ± 0.0022	0.121 M

**Table 4:** Component ablation results across five independent runs. Results are reported as mean ± standard deviation.

Model	SE	Mamba	Parameters	Macro ROC-AUC	Micro ROC-AUC	Macro PR-AUC	Macro F1	Micro F1
**MSCA-Mamba**	✓	✓	121,149	0.8998 ± 0.0022	0.9119 ± 0.0052	0.7651 ± 0.0036	0.7038 ± 0.0045	0.7474 ± 0.0074
**MSCA-Mamba w/o SE**	✗	✓	119,493	0.8997 ± 0.0016	0.9128 ± 0.0031	0.7644 ± 0.0037	0.7012 ± 0.0024	0.7462 ± 0.0044
**Multi-Scale CNN w/o SE/Mamba**	✗	✗	51,141	0.9012 ± 0.0013	0.9175 ± 0.0025	0.7644 ± 0.0019	0.7010 ± 0.0034	0.7492 ± 0.0065
**Single-Scale + SE + Mamba**	✓	✓	114,813	0.8964 ± 0.0011	0.9140 ± 0.0020	0.7579 ± 0.0017	0.6977 ± 0.0029	0.7438 ± 0.0024

**Note:** ✓ indicates that the corresponding component is included, whereas ✗ indicates that it is not included.

**Table 5:** Ablation analysis of alternative SE placements within the MSCA-Mamba architecture (seed = 42).

Conf.	SE Placement	Parameter	Macro ROC-AUC	Micro ROC-AUC	Macro PR-AUC	Macro F1	Micro F1	Weighted F1
**No SE**	None	119,493	0.8986	0.9115	0.7631	0.7016	0.7465	0.7464
**Branch-wise SE (proposed)**	Per branch, before fusion	121,149	0.9020	0.9188	0.7693	0.7100	0.7571	0.7553
**Post-fusion SE**	After feature fusion	121,133	0.9028	0.9185	0.7661	0.7070	0.7519	0.7501
**Post-Mamba SE**	After Mamba	121,133	0.9006	0.9190	0.7639	0.7140	0.7599	0.7586

**Table 6:** Comparison of MSCA-Mamba with parameter-matched sequence-modeling alternatives across five independent runs.

Sequence Model	Parameters	Macro ROC-AUC	Micro ROC-AUC	Macro PR-AUC	Macro F1	Micro F1
**Mamba (proposed)**	121,149	0.8998 ± 0.0022	0.9119 ± 0.0052	0.7651 ± 0.0036	0.7038 ± 0.0045	0.7474 ± 0.0074
**Conv1D**	120,653	0.9043 ± 0.0014	0.9225 ± 0.0018	0.7719 ± 0.0027	0.7094 ± 0.0055	0.7540 ± 0.0075
**GRU**	120,906	0.9062 ± 0.0014	0.9251 ± 0.0010	0.7764 ± 0.0026	0.7115 ± 0.0036	0.7559 ± 0.0024
**LSTM**	120,953	0.9044 ± 0.0010	0.9227 ± 0.0026	0.7706 ± 0.0030	0.7070 ± 0.0018	0.7524 ± 0.0041
**Transformer**	121,405	0.8971 ± 0.0008	0.9161 ± 0.0006	0.7569 ± 0.0018	0.7018 ± 0.0043	0.7484 ± 0.0043

**Table 7:** Comparison of unidirectional and bidirectional Mamba configurations across five independent runs.

Model	Parameters	Macro ROC-AUC	Macro PR-AUC	Macro F1
**Uni-Mamba**	121,149	0.8998 ± 0.0022	0.7651± 0.0036	0.7038 ± 0.0045
**BiMamba**	214,077	0.9047 ± 0.0018	0.7712 ± 0.0038	0.7098 ± 0.0032

**Table 8:** Computational characteristics of MSCA-Mamba and the evaluated sequence-modeling alternatives (batch size = 1).

Model	Parameters	FP32 Model Size (MB)	Latency (ms/Record)	P95 Latency (ms)	Throughput (Records/s)	Peak GPU Memory (MB)
**MSCA-Mamba**	121,149	0.462	2.286 ± 0.033	2.356	437.48	36.40
**MSCA-BiMamba**	214,077	0.817	2.904 ± 0.097	2.998	344.37	37.96
**Matched Conv1D**	120,653	0.460	1.843 ± 0.209	2.164	542.46	35.29
**Matched GRU**	120,906	0.461	2.061 ± 0.061	2.118	485.09	48.49
**Matched LSTM**	120,953	0.461	2.010 ± 0.044	2.066	497.56	50.27
**Matched Transformer**	121,405	0.463	2.037 ± 0.060	2.117	490.97	36.63

## Data Availability

The data used in this study are publicly available in the PTB-XL dataset on PhysioNet: https://physionet.org/content/ptb-xl/1.0.3/ (accessed on 1 June 2026).

## Abbreviations

The following abbreviations are used in this manuscript:

ECG	Electrocardiogram
MSCA-Mamba	Multi-Scale Channel-Attentive Mamba
SE	Squeeze-and-Excitation
CVD	Cardiovascular diseases
AI	Artificial intelligence
CNN	Convolutional Neural Network
DWT	Discrete wavelet transform
SSM	State Space Model
LN	Layer Normalization
GAP	Global Average Pooling
GMP	Global Max Pooling
GRU	Gated Recurrent Unit
LSTM	Long Short-Term Memory
GPU	Graphics Processing Unit
ROC-AUC	Receiver Operating Characteristic Area Under the Curve
PR-AUC	Precision–Recall Area Under the Curve
